# The Role of Next-Generation Sequencing (NGS) in the Relationship between the Intestinal Microbiome and Periprosthetic Joint Infections: A Perspective

**DOI:** 10.3390/antibiotics13100931

**Published:** 2024-10-01

**Authors:** Salvatore Gioitta Iachino, Federica Scaggiante, Claudia Mazzarisi, Christian Schaller

**Affiliations:** 1 Department of Orthopaedics and Traumatology, Hospital of Brixen, Lehrkrankenhaus der Paracelsus Medizinischen Privatuniversität, Südtiroler Sanitätsbetrieb (SABES-ASDAA), 39042 Brixen, Italy; christian.schaller@sabes.it; 2 Laboratory of Clinical Pathology, Hospital of Brixen, Lehrkrankenhaus der Paracelsus Medizinischen Privatuniversität, Südtiroler Sanitätsbetrieb (SABES-ASDAA), 39042 Brixen, Italy; federica.scaggiante@sabes.it; 3 Department of Internal Medicine, Hospital of Brixen, Lehrkrankenhaus der Paracelsus Medizinischen Privatuniversität, Südtiroler Sanitätsbetrieb (SABES-ASDAA), 39042 Brixen, Italy; mazzarisiclaudia@yahoo.it

**Keywords:** gut microbiome, next-generation sequencing (NGS), periprosthetic joint infections (PJIs), CN-PJI, infection

## Abstract

Periprosthetic joint infections are still a challenge in orthopedics and traumatology. Nowadays, genomics comes to the aid of diagnosis and treatment, in addition to traditional methods. Recently, a key role of the intestinal microbiota has been postulated, and great efforts are aimed at discovering its interconnection, which shows to be at different levels. Firstly, the gut microbiome influences the immune system through the gut-associated lymphoid tissue (GALT). A balanced microbiome promotes a strong immune response, which is essential to prevent all local and systemic infections, including PJI. Thus, a dysbiosis, i.e., the disruption of this system, leads to an imbalance between the various strains of microorganisms co-existing in the gut microbiome, which can result in a weakened immune system, increasing susceptibility to infections, including PJI. Additionally, the dysbiosis can result in the production of pro-inflammatory mediators that enter the systemic circulation, creating a state of chronic inflammation that can compromise the immune system’s ability to fend off infections. Furthermore, the microbiome maintains the integrity of the gut barrier, preventing the translocation of harmful bacteria and endotoxins into the bloodstream; dysbiosis can compromise this protective “wall”. In addition, the gut microbiome may harbor antibiotic-resistance genes; during antibiotic treatment for other infections or prophylaxis, these genes may be transferred to pathogenic bacteria, making the treatment of PJI more difficult. In this complex landscape, next-generation sequencing (NGS) technology can play a key role; indeed, it has revolutionized the study of the microbiome, allowing for detailed and comprehensive analysis of microbial communities. It offers insights into the functional potential and metabolic capabilities of the microbiome, studies the collective genome of the microbiome directly from environmental samples sequencing DNA without isolating individual organisms, analyzes the RNA transcripts to understand gene expression and functional activity of the microbiome, analyzes the RNA transcripts to understand gene expression and functional activity of the microbiome, investigates the metabolites produced by the microbiome and studies the entire set of proteins produced by the microbiome. NGS technology, the study of the micromyoma and its implications in the field of orthopedic trauma are innovative topics on which few publications are yet to be found in the international scientific literature. The costs are still high, the focus of research is maximum, and it will certainly change our approach to infections. Our study is an up-to-date review of the hot topic application of NGS in the study and investigation of periprosthetic infections and the microbiome.

## 1. Introduction

Today, total joint arthroplasty is considered one of the three most successful surgeries in the world (along with cataract surgery and cardiac bypass surgery). Most patients forget the operated side after 12 months due to the complete resolution of symptoms and pain-free movement even in low-impact sports.

A periprosthetic joint infection (PJI) is a serious complication of total joint arthroplasty, which can lead to serious consequences (from septic shock to amputation in rare cases) and severe disabilities (pain, ROM limitation), increases healthcare costs and hospitalization time, lengthens recovery time and increases the timeframe of returning to work.

Prosthetic joint infections account in 25% of failed knee arthroplasties and 15% of failed hip arthroplasties, additionally is associated with a 5-year mortality rate higher than that of common cancer tumors. Last but not least is the economic issue: the cost of treating PJI is considerable, estimated at more than $1 billion in 2017 [1].

Worldwide, the number of hip and knee replacements is constantly increasing. In the United States alone there were 332,000 total hip and 719,000 total knee arthroplasties performed in 2010. The numbers are projected to reach 572,000 and 3.48 million by 2030 for hips and knees, respectively [2].

In 2022, in Italy, 125,000 hip prostheses, 100,000 knee prostheses and 13,000 shoulder prostheses were respectively implanted [3].

The longevity of a prosthesis depends on several factors related to the patient and the surgery; a meta-analysis from the University of Britol published in 2019 stated that 58% of patients could expect a hip replacement to last 25 years [4].

Infection represents the most feared complication with potentially serious consequences, the diagnosis is not always simple and the correct flow chart for treatment is, nowadays, still debated. The management of a PJI is complex and expensive, requiring, in most cases, revision surgery and long-term use of antibiotics, and it is associated with significant morbidity and mortality.

## 2. Periprosthetic Joint Infections (PJIs)

The incidence of PJI following hip and knee arthroplasty is estimated at 1% to 2% and still today there is no uniformly accepted definition for this complication [5]. In the current literature, many diagnostic criteria have been proposed, including from the *Musculoskeletal Infection Society* (MSIS), the *International Consensus Meeting* (ICM) for PJI and the *European Bone and Joint Infection Society* (EBJIS). They all share some common criteria but significant variations exist between them.

PJI etiologic agents include bacteria and fungi. *Staphylococcus epidermidis* represents the most frequently isolated pathogen, but *Staphylococcus aureus* and various species of *Streptococcus*, *Enterococcus*, *Cutibacterium* and *Enterobacterales* are also commonly encountered [6]. Approximately, 70% of the PJIs are considered monomicrobial, while 25% are considered polymicrobial [7].

PJI is a complex and challenging complication of joint-replacement surgery that requires a multidisciplinary approach for effective management. Early diagnosis and appropriate treatment are essential to improve outcomes for affected patients.

Ongoing research into better diagnostic tools and treatment options continues to evolve, aiming to reduce the incidence and impact of PJI.

This, for example, was demonstrated in a publication by Prof. Indelli in 2021, which highlighted the recent use of nanoparticles both in the prevention (coating of prosthetic components) to reduce the ability of bacteria to adhere and form biofilm and in the treatment (delivering drugs directly to the infected site) to reduce systemic side effects and increase efficacy [8].

Another recent line of study highlights the state of poor nutrition with susceptibility to periprosthetic infections. Albumin is a protein that is mistakenly considered an indicator of nutrition; in reality, its role is much more complex. Its function is certainly related to the intestinal mucosa with reciprocal interaction: the alteration of the albumin level is an indicator of a systemic state of infection and may negatively modify the permeability of the intestine, as well as its microbiome. Some recent studies have correlated low blood levels to an increased risk of failure and mortality after two-stage revision [9].

PJI remains a current topic not only because of these innovative and “futuristic” lines of research but also because there is no standardized and universally accepted diagnostic and treatment flowchart. Mistakes are still being made in clinical practice, and the article by the authoritative Prof. Trampuz, “Twenty common errors in the diagnosis and treatment of periprosthetic joint infection”, which highlights some of the errors that are likely to be made in practice, for example, partially incorrect diagnostics (synovial leucocyte count is often “forgotten”; ESR and pcr should play a less decisive role), the incorrect use of antibiotic therapy, inadequate swabbing, the non-discriminatory use of pulsatile lavage and incorrect surgical techniques [10].

The connection between intestinal macrobiome and PJI represents the most recent line of research, which is very interesting for its possible clinical and diagnostic implications. Much has already been discovered, but much still remains unclear.

The macrobiome, i.e., the colonies of microorganisms in the gut, has been shown to directly influence the immune system and thus susceptibility or resistance to musculoskeletal infections. Among the various areas, certainly, the one regarding joint prosthesis infections is still a great challenge for the correct treatment, as many variables come into action, and the decision flowchart is not uniform in all cases.

In recent decades, there has been great interest in a type of bacteria and microorganisms that cannot be identified by common laboratory techniques because they are in a state of quiescence but can reactivate and become pathogenic again, i.e., cause infections.

In recent years, it has been shown that the gut is the site of most of these types of microorganisms [11], which we present in the next section.

## 3. Culture-Negative PJI

In this scenario, clinical symptoms are often aspecific (the presence of fever and pus is often lacking), so the diagnosis is mainly based on classical, instrumental laboratory investigations. Unfortunately, there is an increased fear of culture negativity due to intrinsic (i.e., low virulence of the organism or even the presence of viable but not culturable (VBNC) microorganisms) and extrinsic (i.e., early administration of broad-spectrum antibiotics) factors.

VBNC describes a state in which bacteria remain viable but are unable to grow on routine laboratory culture media under conditions normally supportive of their growth [11]. This state is often induced by various environmental stresses, such as nutrient limitation, temperature extremes, pH changes or exposure to antibiotics; indeed, the inappropriate use of antibiotics, due to misdiagnosis or administration of incorrect doses, can lead to a VBNC state in bacteria.

This state of “quiescence”, a condition well described in cell biology, can last for several months and is also reversible; this means that bacteria can regain their infectious capacity, reacquire their metabolic properties, create an active infection and thus become culturable again.

*Katja Šuster* and *Andrej Cör* succeeded in inducing the VBNC state in certain bacteria (*S. epidermidis*, *S. lugdunensis* and *S. aureus*) by subjecting them to stress conditions and different concentrations of antibiotic (gentamicin); in the experiment, the bacteria were then identified using Bacteriophages and qPCR [12].

This distinctive condition by which bacteria can temporarily “deactivate” themselves was first described in 1982 by Xu et al. on certain strains of Gram-negative bacteria (*Escherichia coli* and *Vibrio cholera*). To date, it has been shown that several bacterial types, more than 100, can acquire this condition.

While studies were initially based on cell-staining procedures, other nucleic acid-based methods have recently been developed that allow the detecting and especially the marking of the vitality of cellular microorganisms. It is important to specify that it is not possible to distinguish between dead and live bacteria using the traditional PCR or qPCR technique, as these methods only target DNA.

To solve this problem, a recent technique has been developed using ethidium monoazide (EMA) and propidium monoazide (PMA) in real-time PCR to measure the DNA of live cells. EMA and PMA get through the damaged cell membrane, enter the dead bacteria and react with the hydrocarbon portion of the DNA of the dead cells, causing structural changes in the DNA. With these changes, the DNA of dead cells cannot replicate in the PCR reaction, and only the DNA of live cells can replicate.

Another method based on molecular diagnostic approaches is quantitative reverse-transcription PCR (RT-qPCR), which is based on mRNA. Another recent technique used to detect VBNC cells is the use of biosensors, i.e., a phage-based biochip using magnetic nanoparticles.

Despite countless studies since 1982 on the subject, especially in the field of public health and biotechnology, *Darcan* et al. [13] conclude that “the mechanism of the VBNC state still contains serious loopholes and VBNC poses a problem for public health, as cells retain their virulence and can resuscitation under appropriate conditions”. The phenomenon of reanimation from the VBNC state was first recognized in *Salmonella enteritidis* and *E. coli* in 1984 and consists of the transition, under favorable conditions, to an “active”, i.e., cultivable, state [12]. As mentioned, VNBC bacteria are not detected by common culture media for bacterial growth. 

A comprehensive definition of “culture-negative periprosthetic infections” (CN-PJIs) was formulated by *Berbari* [14], who defines it as “the failure of bacterial growth in aerobic or anaerobic cultures from intra-operative tissue biopsy samples from the periprosthetic area in patients with the presence of purulent fluid and/or with signs and symptoms of acute inflammation or with a sinus tract communicating with the implant”.

The pathogens involved in CN-PJI are caused by fungi and mycobacteria in more than 85% of all cases; the remaining 25% are caused by bacteria, and of these, more than 50% are sustained *Brucella* and *Coxiella burnetii*.

In the case of periprosthetic infections with negative culture examinations prior to surgical and pharmacological treatment, it is mandatory to distinguish whether it is a true negative (i.e., we have a case of aseptic loosening) or a false negative (i.e., the culture failed to identify the organism, which is actually the cause of the prosthetic failure). It is interesting to observe, in this regard, that the literature does not agree on the exact prevalence of culture-negative PJI; in fact, the various scientific papers on the subject report data ranging from 5% to 42%.

Risk factors that may facilitate the development of CN-PJI are similar to those for culture-positive PJI and include obesity, malnutrition, advanced age and co-morbidities (chronic kidney disease, diabetes mellitus, rheumatoid arthritis, liver disease and vascular insufficiency); obesity with a BMI over 40 is, in particular, a strong risk factor for PJI.

CN-PJI also has specific risk factors, i.e., a previous history of PJI or surgical site infection (SSI), previous revision surgery and previous long-term use of antibiotics [13,14].

Identification of the pathogen is the key to guiding targeted antibiotic therapy. However, there is currently no gold standard for the definitive diagnosis of CN-PJI, which requires a combination of clinical evaluation, radiological instrumental examinations (standard radiography, CT, MRI with metal artefact suppression sequences, scintigraphy, etc.), serological (serological and synovial inflammatory markers), histopathological (intraoperative biopsy samples) and microbiological (cultural examination on pre-operative, intraoperative and/or sonication samples).

Synovial biomarkers are increasingly playing an important role in diagnosis: glucose and leucocyte esterase levels, in particular, have proven to be reliable and accurate in terms of sensitivity and scientificity [15].

The most commonly used serological markers in clinical practice are C-reactive protein (CRP) and the erythrocyte sedimentation rate (ESR): for early PJI, a CRP value of 10 mg/L is the threshold, and for ESR, >30 mm/h [16].

The reliability (sensitivity and specificity) of the many serum markers is not yet agreed upon in the scientific literature; several studies report conflicting data.

ESR and CRP remain the most reliable values, although sensitivity and specificity do not reach 100%.

For the correct use and interpretation, it must be taken into consideration that the CRP level increases after surgery, reaching a maximum level on the second day after surgery and returning to preoperative levels after three weeks; the ESR reaches a maximum level on the fifth day after surgery and remains elevated up to 12 months after surgery.

They are non-specific for infections and may be elevated for other reasons, such as autoimmune diseases and infections of other origins. The sensitivity for CRP is 88%, with a specificity of 74%. The sensitivity for ESR is lower, at 75%, with a specificity of 70%. Ultimately, a normal CRP value (i.e., <10 mg/L) and a normal ESR (i.e., <30 mm/h) rule out PJI with a sensitivity of 96%.

Other serological markers studied and used in recent years are leucocyte esterase, alpha-defensin, D-dimer and interleukin-6 (IL-6) [15,16]. Leucocyte esterase (LE) is an enzyme secreted by neutrophils in the presence of infection. Parvizi et al. first described its potential role in the diagnosis of PJI in 2011 (and then confirming in the 2018 study), demonstrating a specificity and sensitivity of 100% and 81%, respectively. The authors concluded that LE is a low-cost screening tool and that its specificity has proven accurate in excluding cases of infection.

The alpha-defensin protein is an antimicrobial peptide released by neutrophils in response to a pathogen in synovial fluid. Alpha-defensin tests have been shown to have a high sensitivity (97%), high specificity (97%), high positive predictive value (88%) and negative predictive value (99%). D-dimers are fibrin degradation products that are formed when a fibrin clot is dissolved by plasmin. Although the blood value may increase under different conditions, it has a high sensitivity and specificity in the diagnosis of PJI, especially in the early phase. D-dimer is an inexpensive and easy test to perform: an immediate increase in levels after surgery followed by an abrupt return to normal offers the promise of using D-dimer as a marker for PJI in both early and late phases.

Another recently studied marker is interleukin-6, which is produced in vivo by cells in the inflammatory response of the lymphatic system following trauma, infection and surgery; recent meta-analyses have demonstrated its efficacy and reliability.

In addition to serological markers, a few other culture-based methods have been proposed to improve the identification rate, including Sonication and Dithiothreitol analysis [16].

Recently, innovative next-generation sequencing (NGS) technologies, which allow for a genomically based microorganism identification, have been applied in PJI culture-negative scenarios, especially when “traditional” microbiological techniques have failed.

In the COVID-19 era, NGS has revolutionized genetics in general and PJI diagnostics in particular, due to the capability of identifying the allowing bacterial or fungal genome in a very short time. In a recent study on 86 anonymized synovial fluid samples, the use of NGS was useful in detecting the PJI responsible pathogen with a high (>96%) concordance rate when compared to routine microbiological culture, especially in CN-PJI scenarios [14].

## 4. Next-Generation Sequencing and Metagenomic Next-Generation Sequencing

Next-generation sequencing (NGS) is a method of sequencing deoxyribonucleic acid (DNA) found in a sample from the host or microorganisms, thus allowing for the identification of microorganisms. The technique may be particularly useful when there is a strong clinical suspicion of PJI and when cultures or other diagnostic tests are negative.

Metagenomic next-generation sequencing (mNGS), an evolution of NGS, is able to detect the entire DNA or RNA sequence from a sample, an aspect that allows for the determination of a pathogen of any type (fungi, parasites, bacteria and viruses) in a 24-to-48 h timeframe, which is significantly shorter if compared to the 7–10 days needed for a standard culture analysis.

Metagenomic NGS is a sequencing technique that has a higher yield in respect to NGS, being able to sequence not only the complete bacterial genome but also the antimicrobial resistance genes. Another theoretical advantage of mNGS compared to conventional NGS is represented by the speed of bacterial identification, which has an impact on the initiation of targeted and non-empirical antibiotic therapy and helps to avoid possible secondary toxicities. The reported sensitivity and specificity of mNGS are high, but not 100%, due to the large amount of host DNA usually present in the synovial fluid, which interferes with the bacterial DNA identification [17]. Indeed, although NGS can also detect low-virulence bacteria, contamination of collected samples leading to false positivity remains a challenge.

Then, the combination of mNGS and classic microbiological techniques for bacterial identification could lead to an optimization of the PJI diagnostic rate [14,17].

Unfortunately, the relatively high cost of NGS, compared to standard microbiological tests, may represent a limitation of its current use, but this must be weighed against the final cost of an infected arthroplasty in which the germs cannot be identified and the ineffectiveness of empiric antibiotic therapy.

Another important aspect that must be discussed is the ability of NGS to detect a potential underlying “native” joint microbial community. For a long time, it was believed that human joints were characterized by having a sterile environment, but the recent literature highlighted the existence of an endemic, transitory microbiome in the human joints. This research is in its early stages but will certainly be another international hot topic in the near future [18].

With regard to microbiome research, the applications of NGS cover many different fields of microbiological science, and on each of them, it can play a central role [17,19]:

1. Gene sequencing (the science of detecting the sequence of nucleotides in DNA and RNA):
  - Identifies and classifies bacteria based on the 16S ribosomal RNA gene, which is highly conserved among different species of bacteria.
  - Amplification and sequencing of the 16S rRNA gene, followed by a comparison to reference databases.
  - Technique that showed rapid and cost-effective profiling of bacterial communities.
2. Whole-genome sequencing WGS (the laboratory process of determining the entirety of the DNA sequence of an organism’s genome at a single time, i.e., 3 billion nucleotides):
  - Provides comprehensive information about the entire genetic material of microorganisms within a sample.
  - Sequencing all the DNA in a sample, including bacteria, viruses and fungi.
  - Offers detailed insights into the functional potential and metabolic capabilities of the microbiome.
3. Metagenomics (research discipline that uses molecular biology methods to acquire, analyze and interpret sequence data of whole biological communities from a particular habitat):
  - Studies the collective genome of the microbiome directly from environmental samples.
  - Sequencing DNA extracted from a sample without isolating individual organisms.
  - Captures the diversity and complexity of microbial communities, including unculturable microorganisms.
4. Metatranscriptomics (set of techniques used to study gene expression of microbes within natural environments):
  - Analyzes the RNA transcripts to understand gene expression and functional activity of the microbiome.
  - Sequencing RNA extracted from a sample, often after converting it to cDNA.
  - Provides insights into active biological processes and microbial interactions.
5. Metaproteomics (qualitative and quantitative protein analysis in microbial communities and microbiomes from environmental sources):
  - Studies the entire set of proteins produced by the microbiome.
  - Mass spectrometry and other proteomic techniques to identify and quantify proteins.
  - Reveals functional protein expression and microbial activity at the protein level.
6. Metabolomics (the study of the entire set of metabolites within organism or biological sample:
  - Investigates the metabolites produced by the microbiome.
  - Analytical techniques such as mass spectrometry or NMR spectroscopy to profile small molecules.
  - Provides insights into the metabolic functions and interactions within the microbiome

NGS generates large and complex datasets requiring robust bioinformatics tools and expertise for analysis; indeed, while costs have decreased, NGS can still be expensive and resource-intensive.

Despite the great potential of NGS, linking microbial composition to functional outcomes and clinical relevance can be challenging [17,19].

## 5. Gut Microbiota and Microbiome

The human microbiota is defined as the set of microorganisms that, physiologically, or sometimes pathologically, lives in symbiosis with the human body; the microbial population is mainly concentrated in the gut.

The gut microbiota refers to the complex community of microorganisms, including bacteria, viruses, fungi and other microbes, residing in the gastrointestinal tract; the approximate number of bacteria composing the gut microbiota is about 10^13^–10^14^. The term “microbiome” indicates, instead, the totality of the genetic heritage possessed by the microbiota (i.e., the genes which the microbiota is able to express).

The relationship between the gut microbiota and various aspects of human health, including the immune system and inflammatory responses, has been a topic of increasing interest in medical research [20].

The connection between the gut microbiota and PJIs is an emerging area of research, and NGS plays a crucial role in advancing orthopedic surgeons’ understanding of this matter.

Understanding the role of the gut microbiome in PJI through NGS could have implications for preventive strategies, such as preoperative screening and interventions to modulate the gut microbiome to reduce the risk of infection. However, it is important to note that research in this area is still evolving, and more studies are needed to establish clear associations and mechanisms [21].

NGS can have several roles in this field: Microbial Profiling (identify the composition and amount of bacteria in the gut microbiome), Metagenomics (understand how the gut microbiome influences the host’s health and studying its functionality), Microbiome Dysbiosis (identify an imbalance or disruption in the normal composition of the gut microbiome that may be associated with inflammatory conditions and immune system dysregulation, which could contribute to the risk of PJI) and, finally, Biomarker Discovery (identify specific genetic markers released by the bacteria that are associated with an increased or decreased risk of PJI) [21,22].

There are several sequencing-based approaches that can provide useful information for a better understanding of the role of the gut microbiome in the maintenance of homeostasis and disease onset. The various sequencing approaches are not in conflict with each other and could be integrated for a comprehensive understanding.

In addition, other techniques, such as metaproteomics and metabolomics, could contribute to the understanding of the complex function of the gut microbiota [22,23].

A recent study by *Hernandez* et al. (2019), using an animal model, showed that the destruction of the gut microbiota with oral antibiotic therapy tripled the risk of PJI [24] and also suggest to perform randomized clinical trials to study whether the introduction of probiotics in oral therapy can modulate the immune system to reduce PJI risk and even reduce nasal colonization of pathogens such as MRSA [24].

**Figure 1:**
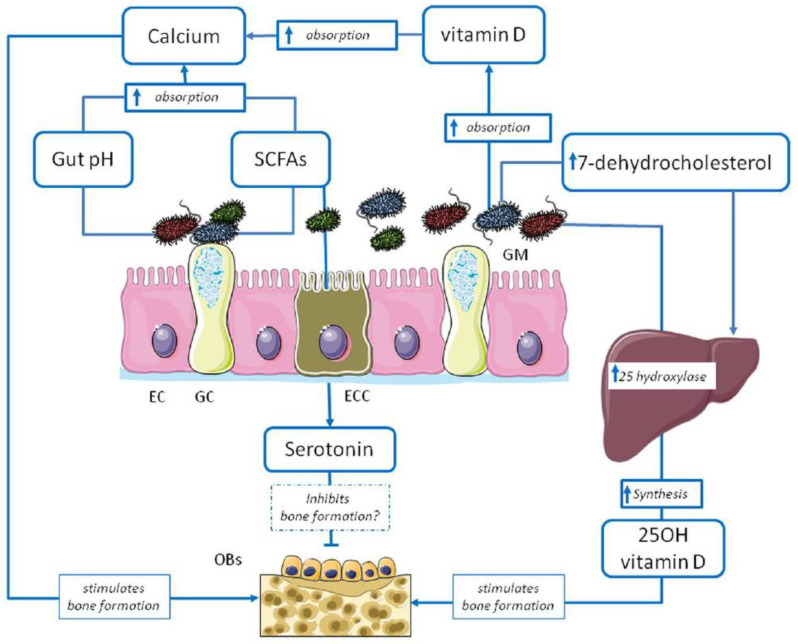
The design demonstrates the relationship between gut microbiota and bone metabolism.

Several studies have confirmed that the complex gut microbial network directly and indirectly (e.g., production of metabolites) influences bone metabolism and the homeostats of calcium, hormones and vitamins [23], while the 16S rRNA gene sequencing demonstrated dysbiosis in osteoporosis [25]. Figure 1 simplifies the close connection between intestinal macrobiome and metabolism.

A study from the University of Bordeaux showed that antibiotic therapy alters the composition and quantity of the microbiota; these modifications were associated with a quantitative increase in markers of mucosal inflammation, gut permeability and, finally, elevated levels of CRP [26].

Preclinical studies in the laboratory have shown that *Staphylococcus aureus*, one of the most common causes of PJI, is able to translocate from the gut to prosthetic joints or to the surgical site SSI (surgical site infection). In this regard, the “Trojan horse” theory has recently been formulated, according to which the translocation process does not only occur via the blood (i.e., through bacteremia), but also by blood cells such as neutrophils and macrophages acting as a “Trojan horse” transporting pathogens between various sites [27].

In connection with this, it is interesting to mention Moschetti’s article again that published a study where 40 patients, on the day of arthroplasty surgery, underwent joint arthrocentesis and biopsy sampling for NGS analysis; the result showed that, in 30% of the patients, at least one microorganism was present in the intra-articular space, and of these patients, 75% showed several microorganisms simultaneously [18].

Several studies confirm that antibiotics have a great negative impact on the intestinal microbiota and thus on the patient’s immune capacity; this process is, however, reversible, with a rapid but partial recovery observed 2 weeks after stopping antibiotics. It is therefore essential to minimize antibiotic use, preferably by using in situ antibiotics or using antibiotics with a narrow spectrum to limit the impact on homeostasis.

However, in many infections, antibiotics cannot be substituted, and long-term high-dose systemic treatment is mandatory to cure patients; in these situations, probiotic-based drug therapy, to counteract the deleterious effects of such treatments, could be important, especially in elderly patients and those with other chronic diseases.

Nowadays, microbiota transplantation is the only decisive treatment that allows for the grafting of a complex ecosystem with proven functional benefits; it consists of transferring the fecal microbial ecosystem of a healthy donor to a recipient to restore intestinal homeostasis. The human gut microbiome, represented by trillions of microorganisms colonized in the human gut, is a major contributor to human metabolism and health; the microbiota localized in the gastrointestinal tract is capable of performing multiple roles for the human host, including nutritional, physiological, and immunological functions.

It is therefore closely linked to the body’s ability to react/resist to periprosthetic infections. Historically, gut microbiome studies have been restricted due to the difficulties in culturing many of these gut microbial species in laboratory conditions.

The development of metagenomics based on NGS has made it possible to avoid the traditional bias of culture dependence and has significantly extended our understanding of the composition, diversity and role of the gut microbiome in human health and disease [21,22,24].

Therefore, several recent preclinical laboratory studies have supported a close relationship between pathogens and the immune system, but confirmation of this phenomenon in vivo remains unproven.

## 6. Conclusions

Periprosthetic infections represent one of the main challenges in orthopedics, leading to a huge healthcare cost and impacting heavily on patients’ quality of life.

Many research efforts have improved treatment, prevention and, above all, diagnosis. Indeed, one of the main problems in the correct diagnosis and treatment of a periprosthetic infection is represented by the accurate identification of the responsible agent.

In recent years, scientific research has developed NGS technologies that guarantee rapid, reliable identification of the responsible pathogen and also independent of antibiotic therapy. NGS is a rapid method of parallel sequencing of deoxyribonucleic acid; the technique can be particularly useful when there is a strong clinical suspicion of PJI despite the culture examination or other diagnostic tests being negative. The sensitivity and specificity of mNGS are high, their potential is enormous, and in recent years, they have increasingly played a major role [28].

These modern technologies cannot, however, exclude “traditional” techniques; on the contrary, they require their “alliance”. Indeed, the combination of mNGS with classical microbiological techniques for bacterial identification thus leads to an ideal optimization of the diagnosis and treatment of periprosthetic infections.

The connection between intestinal microbiota and periprosthetic infection has recently been postulated, a pioneering topic that could revolutionize current notions and improve our prevention and treatment capabilities.

The possible implications are not yet fully known and probably not even foreseeable, but it will certainly change our future approach to this crucial topic.

Despite the progress that research has already made, randomized and multicenter studies are needed to improve the surgeon’s knowledge of these innovative technologies.

## Data Availability

Not applicable.

## Abbreviations

gut microbiota (GM), enteral cells (EC), enterochromaffin cells (ECC), osteoblasts (OBs), goblet cells (GC), Short Chain Fatty Acid (SCFas).
